# Neoadjuvant Treatment of Stage IIB/III Triple Negative Breast Cancer with Cyclophosphamide, Doxorubicin, and Cisplatin (CAP Regimen): A Single Arm, Single Center Phase II Study (GBECAM 2008/02)

**DOI:** 10.3389/fonc.2017.00329

**Published:** 2018-01-24

**Authors:** Arlindo R. Ferreira, Otto Metzger-Filho, Roberta M. B. Sarmento, José Bines

**Affiliations:** ^1^Department of Medical Oncology, Hospital de Santa Maria, Instituto de Medicina Molecular, Faculdade de Medicina, Universidade de Lisboa, Lisbon, Portugal; ^2^Department of Medical Oncology, Dana Farber Cancer Institute, Boston, MA, United States; ^3^Department of Medical Oncology, Instituto Nacional de Câncer, Rio de Janeiro, Brazil

**Keywords:** breast cancer, triple negative, neoadjuvant chemotherapy, locally advanced cancer, cisplatin

## Abstract

**Background:**

The DNA damaging platinum salts have been explored in the treatment of triple negative breast cancer (TNBC) based on preclinical, and, more recently, clinical evidence of specific susceptibility of TNBC to these agents. Despite the increased toxicity, treatment intensification with polychemotherapy improves response and might be of interest in patients presenting with large primaries. In this trial, we aimed at exploring the efficacy and tolerability of the addition of cisplatin to standard anthracycline–cyclophosphamide backbone in patients with stage IIB/III TNBC.

**Patients and methods:**

This is a single arm, single center, non-randomized, phase II trial of stage IIB/III TNBC. Patients received neoadjuvant chemotherapy with cisplatin (50 mg/m^2^) in combination with doxorubicin (50 mg/m^2^) and cyclophosphamide (500 mg/m^2^) every 21 days and for a total of six cycles (CAP). After surgery, adjuvant chemotherapy consisting of docetaxel (75 mg/m^2^) every 21 days was further provided for four cycles. Primary outcome was pathological complete response in the breast and axilla (pCR; ypT0ypN0). Secondary outcomes were safety, disease-free survival (DFS), and overall survival (OS).

**Results:**

Eight (19.5%) out of 41 patients reached a pCR and 35 (85.4%) had a clinical complete or partial response. After a median follow-up of 47.4 months (interquartile range 30.9–61.9), the proportion of patients free of recurrence or death at 3 years was of 51.8% [95% confidence interval (CI) 34.6–66.5%], while the proportion of patients alive at 3 years was of 55.5% (95% CI 37.8–70.1%). Patients with a pCR rate or family history of breast and/or ovarian cancer showed a numerical but statistically non-significant trend for improved DFS and OS. The majority of patients received six cycles of CAP (82.9%). The three most common grade ≥3 adverse events were nausea (16.3%), vomiting (14.0%), and neutropenia (9.3%). Febrile neutropenia occurred in three patients (7.0%).

**Conclusion:**

Cisplatin in association with doxorubicin and cyclophosphamide was associated with a pCR rate of 19.5% in a cohort of patients with predominantly stage III tumors. The tolerability profile of this combination poses clinical challenges to its general use in clinical practice.

**Unique Identifier Number:**

GBECAM 2008/02.

**NCT Identifier Number:**

NCT03304756.

## Introduction

Triple negative breast cancer (TNBC) is a clinical group of breast invasive tumors that account for approximately 15–20% of all breast cancers, both worldwide and in Brazil (1, 2). This group of tumors is clinically defined as both the absence of estrogen and progesterone receptors plus the absence of HER2 overexpression/amplification. Clinically, TNBC tends to present as fast growing tumors, often diagnosed as locally advanced.

Taken as a group, locally advanced breast cancers (LABC), generally defined as stage III tumors, account worldwide for about 10–30% of all breast cancers (3, 4). In some settings, as in Brazil, the proportion of patients with LABC is in the upper estimate, with approximately 25% of breast cancers presenting with stage III disease (and up to 47% with stage II disease) (2). The conjunction of TNBC biology and locally advanced presentation poses a clinical challenge: TNBC presents a high response to chemotherapy, but the likelihood of relapse remains high (5, 6). Therefore, systemic therapy before surgery (primary systemic therapy) is the most appropriate approach for patients with LABC, especially of TNBC biology, given the improved rate of inoperable tumors that become subsequently operable, the proportion of patients not candidates to breast-conserving surgery that are afterward amenable to receive such procedure, but also given the prognostic implications of the complete response to neoadjuvant chemotherapy, referred as pathological complete response (pCR). In fact, pCR emerged as a strong marker of prognosis, as patients attaining a complete response to primary systemic therapy have improved outcomes both in terms of recurrence and survival (7). This led both the Food and Drug Administration and the European Medicines Agency to recognize the neoadjuvant setting and the outcome pCR as an arena for the evaluation of the performance of different treatment regimens. Thus, in light of the high risk of relapse of TNBC and locally advanced staging, trials aiming at improving the efficacy of neoadjuvant treatments are a clear unmet need and an evolving field in current clinical practice.

In this setting, DNA cross-linking platinum salts [side with poly (ADP-ribose) polymerase—PARP—inhibitors in the metastatic setting] are being used as strategies to improve outcomes in TNBC (8, 9). These tumors present a high mutation load and features of genomic instability that are frequently associated with homologous recombination deficiencies (the most frequent cause being BRCA1 deficiency) (10, 11). The use of platinum salts aims at exploiting homologous recombination deficiency by further challenging this set of pathways in order to achieve synthetic lethality (12). In fact, several studies have now tested platinum salts (both carboplatin and cisplatin) in the neoadjuvant and palliative setting in TNBC (8, 13–16). Data on the use of cisplatin is, however, scarce. In a trial that enrolled 28 patients with TNBC treated with neoadjuvant single agent cisplatin for four cycles, 6 (22%) of 28 achieved a pCR, with 18 (64%) attaining a clinical complete or partial response. Cisplatin in combination with doxorubicin and cyclophosphamide (CAP) was already shown to be an effective and safe regimen to treat ovarian cancer (17–19). Its efficacy in patients with breast cancer is, however, unknown, but CAP regimen is an appealing approach given its standard anthracycline–cyclophosphamide backbone. Moreover, despite the risks for increased toxicity, the combined use of the three agents might further improve response and the proportion of patients reaching a pCR.

In this prospective, single arm, phase II study, we sought to evaluate the efficacy of a neoadjuvant chemotherapy treatment regimen containing cisplatin, doxorubicin, and cyclophosphamide (CAP regimen) followed by adjuvant docetaxel in a population of locally advanced (stage IIB/III) TNBC.

## Patients and Methods

### Study Participants

Eligible patients were women with previously untreated, clinical stage IIB/III breast cancer that was negative for estrogen and progesterone receptors by immunohistochemistry (IHC; <10% staining) and with no HER2 overexpression/amplification by IHC (from 0 to 2+) or *in situ* hybridization (in cases IHC-defined as 2+). Moreover, only patients with an Eastern Cooperative Oncology Group Performance Status ≤2, measurable disease, appropriate hematologic, liver, renal, cardiac functions, and able to follow the protocol were considered as eligible.

### Study Design and Treatment Protocol

This is a non-randomized, open-label, single arm, single center, phase II clinical trial. Patients received neoadjuvant chemotherapy with cisplatin [50 mg/m^2^, intravenous (IV), day 1] in combination with doxorubicin (50 mg/m^2^, IV, day 1), and cyclophosphamide (500 mg/m^2^, IV, day 1) every 21 days for a total of six cycles (CAP regimen). Subsequent mastectomy plus axillary lymph node dissection was performed. Pathological specimen was analyzed to assess tumor response in the breast and axilla. Adjuvant chemotherapy consisting of docetaxel (75 mg/m^2^, IV) every 21 days for four cycles was strongly recommended even in the setting of a pCR, but not mandatory for trial participation nor for inclusion in outcomes assessment. Docetaxel was provided adjuvantly so that the efficacy of CAP in terms of the outcome pCR could be clearly interpreted. In case of tumor progression during neoadjuvant treatment, CAP was discontinued and additional local or systemic treatment was provided at the discretion of the investigator. Given the estimated risk of febrile neutropenia <20%, use of colony-stimulating factors was not mandatory, but was allowed at the discretion of the treating physician.

Baseline breast and axilla assessment were performed clinically and by mammography. Staging procedures included chest X-ray, abdominal ultrasound, bone scan, and bone X-ray in case of suspicious findings on bone scan. Patients were followed every 3 weeks during chemotherapy, every 3–4 months for the first 2 years, every 6 months until year 5, and annually thereafter. Annual mammogram was recommended and further imaging assessments were performed as clinically indicated.

**Figure 1:**
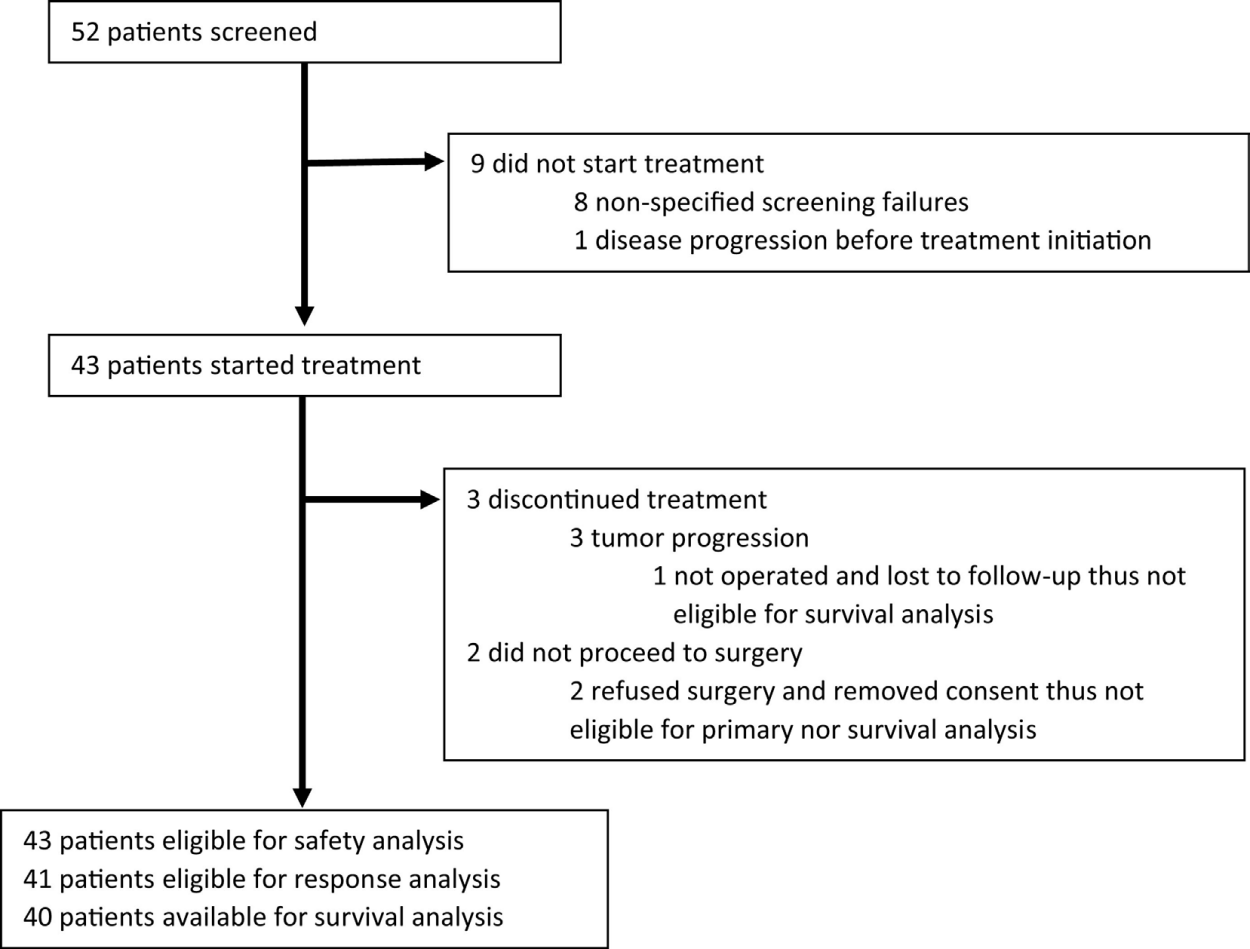
Patients flowchart.

All patients starting CAP regimen were eligible for the safety analysis. For primary and survival analyses, fewer patients were included, as detailed in Figure 1. Patients removing consent before surgery were not eligible for primary and survival analyses and patients lost to follow-up immediately after surgery (in one case missing to follow-up appointment due to change in city of residence and center of care) were not eligible for survival analyses.

The protocol was approved by the institutional review board of *Instituto Nacional de Câncer*. All patients provided written informed consent. The *Grupo Brasileiro de Estudos do Câncer de Mama (GBECAM)* and *Instituto Nacional de Câncer* were the academic sponsors and *Instituto Nacional do Câncer* was the funding source of the trial. This trial is registered at GBECAM with identifier 2008/02 and at clinicaltrials.gov with identifier NCT03304756.

### Outcomes Definition

Primary outcome was pCR as assessed by the local pathology lab, and defined as the absence of tumor (invasive and/or *in situ*) both in the breast and axilla (ypT0 ypN0). Secondary objectives were safety, disease-free survival (DFS), and overall survival (OS). The study was not powered to assess DFS and OS, and therefore, these outcomes are exploratory in nature. Safety was assessed according to NCI CTCAE (National Cancer Institute Common Terminology Criteria for Adverse Events) version 3.0. DFS was defined as time from surgery to disease recurrence or death from any cause, while OS was defined as time from surgery to death from any cause. Family history of breast and/or ovarian cancer (FHBOC) was defined as first-, second-, or third-degree relatives with breast cancer diagnosed ≤50 years, and/or invasive ovarian, fallopian tube, or primary peritoneal cancer at any age.

### Statistical Analysis

Simon’s two-stage optimum design was used. The null hypothesis that the true response rate is 5% was tested against a one-sided alternative. In the first stage, 21 patients were accrued. A minimum of 1 pCR event out of 21 was needed to further proceed with the study. In the second stage, 20 additional patients were further accrued for a total of 41 patients. The null hypothesis would be rejected if four or more extra responses (thus five or more in the overall cohort) were observed in 41 patients. This design yields a type I error rate of 5% and power of 70%, when the true response rate is 15%. The estimated pCR was lower than in historical cohorts of unselected TNBCs due to the advanced nature of the tumors and the fact that the taxane was provided only adjuvantly.

Descriptive statistics of baseline demographic, clinicopathological, and treatment characteristics were performed. Time to event outcomes were estimated and plotted using the Kaplan–Meier method. An exploratory analysis of survival outcomes (DFS and OS) according to pCR status and FHBOC was performed. Survival rates were compared using univariate Cox proportional hazards models. Follow-up was calculated has the median follow-up for censored patients. All tests were 2-sided and *p*-values of ≤0.05 were considered statistically significant. The analyses were performed using Stata 13.1 (StataCorp LP).

## Results

### Patient Characteristics

From December 2007 to April 2012, 43 patients started treatment with CAP. Of these, all were subsequently eligible for surgery; however, two refused to receive the procedure, thus 41 patients were eligible for primary analysis (Figure 1). Patients’ characteristics are listed in Table 1. Median age at study enrollment was 48 years (range 27–83) and the majority of patients (63.4%) had an ECOG performance status of 0. The median tumor size by physical examination was 80 mm [interquartile range (IQR 70–100)]. Moreover, 52.6% of patients presented with cT4 disease and 78.9% presented with lymph node involvement. Fifteen patients (38.5%) had a FHBOC.

**Table 1:** Patients’ demographics and tumor characteristics at baseline.

	Patients undergoing CAP (*n* = 41)
**Age (years), ***n*** (%)**	
<35	15 (36.6)
35–<50	16 (39.0)
50–<70	7 (17.1)
≥70	3 (7.3)
Median (IQR)	48 (38–58)
Min.–Max.	27–83
**PS ECOG, *n* (%)**	
0	26 (63.4)
1	15 (36.6)
**Menopausal status, *n* (%)**	
Premenopausal	16 (39.0)
Postmenopausal	18 (43.9)
Unknown	7 (17.1)
**Clinical T status, *n* (%)**	
T2	1 (2.4)
T3	18 (43.9)
T4	22 (53.7)
**Clinical N status, *n* (%)**	
N0	9 (22.0)
N1	19 (46.3)
N2	11 (26.8)
N3	2 (4.9)
**Clinical TNM staging, *n* (%)**	
IIB	4 (9.8)
IIIA	15 (36.6)
IIIB	20 (48.8)
IIIC	2 (4.9)
**Initial tumor size (mm) on physical exam, *n* (%)**	
≤50	3 (7.3)
>50–≤70	7 (17.1)
>70–≤100	10 (24.4)
>100	21 (51.2)
Median (IQR)	80 (70–100)
**Family history of breast or ovarian cancer, *n* (%)**	
Yes	15 (38.5)
No	24 (61.5)
Unknown	2 (4.9)

*ECOG, Eastern cooperative Oncology Group; IQR, Interquartile range*.

### Treatment Response

Of the 41 patients eligible for primary analysis, 35 (85.4%) had a disease response (Table 2). Of these, 8 [19.5%, 95% confidence interval (CI) 9.8–35.2%] had a pCR (ypT0ypN0) and 9 (22.0%; 95% CI 11.5–37.8%) a pCR in the breast. Four patients had disease progression. For detailed final clinical and pathological tumor size see Table S1 in Supplementary Material.

**Table 2:** Pathologic complete response and response in the breast.

	Patients receiving CAP (*n* = 41)
**Pathological complete response rate, *n* [%; 95% confidence interval (CI)]**	
Breast plus axilla plus no DCIS	8 (19.5; 9.8–35.2)
**Response in the breast, *n* (%; 95% CI)**	
Complete response	9 (22.0; 11.5–37.8)
Partial response	26 (63.4; 47.2–77.1)
Stable disease	2 (4.9; 1.1–18.4)
Disease progression	4 (9.8; 3.6–24.1)

### Survival Outcomes

**Figure 2:**
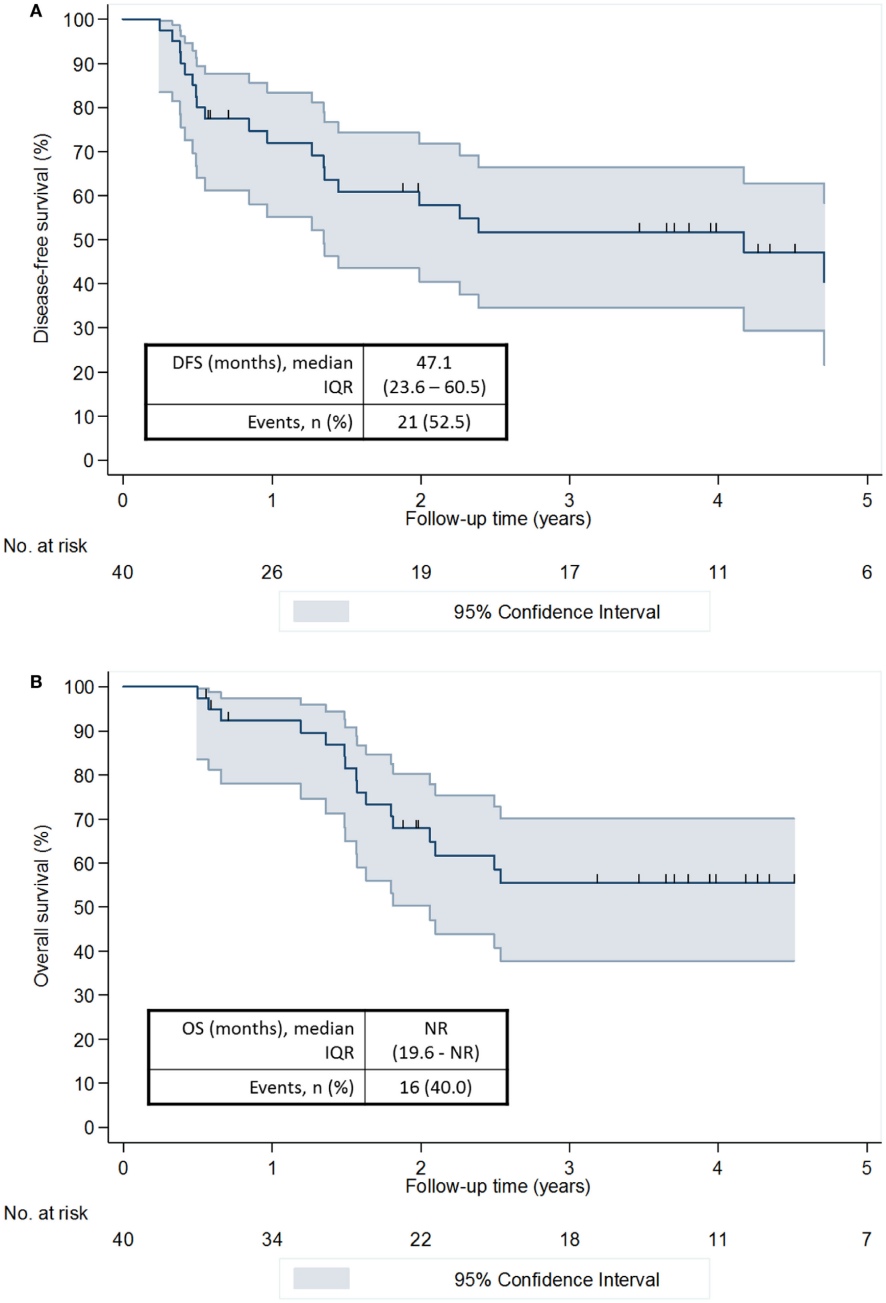
Disease-free survival **(A)** and overall survival **(B)**.

After a median follow-up of 47.4 months (IQR 30.9–61.9; min.–max. 6.7–79.9), 21 (52.5%) recurrences or deaths were recorded (Table 3). The proportion of patients free of recurrence or death at 1 and 3 years was 71.9% (95% CI 54.9–83.4%) and 51.8% (95% CI 34.6–66.5%), respectively (Figure 2A). Median DFS was 47.1 months (IQR 23.6–60.5). In parallel, 14 (36.8%) deaths were recorded. The proportion of patients alive at 1 and 3 years was 92.3% (95% CI 78.0–97.5%) and 55.5% (95% CI 37.8–70.1%), respectively (Figure 2B). Median OS was not reached (IQR 19.6–not reached).

**Table 3:** Recurrence and death events. In case of multiple sites of disease at recurrence, the assigned site of recurrence is the one with worse prognostic implications.

	Patients receiving CAP (*n* = 40)
**Recurrence, *n* (%)**	
Overall	20 (50.0)
**Type of recurrence**	
Locoregional	5 (25.0)
Distant	15 (75.0)
**Site specific**	
Lung	6 (30.0)
Brain	4 (20.0)
Ipsilateral chest wall	3 (15.0)
Regional nodes	2 (10.0)
Contralateral chest wall	2 (10.0)
Distant nodes	1 (5.0)
Bone	1 (5.0)
Liver	1 (5.0)
**Death, *n* (%)**	
Yes	16 (40.0)
No	24 (60.0)

**Figure 3:**
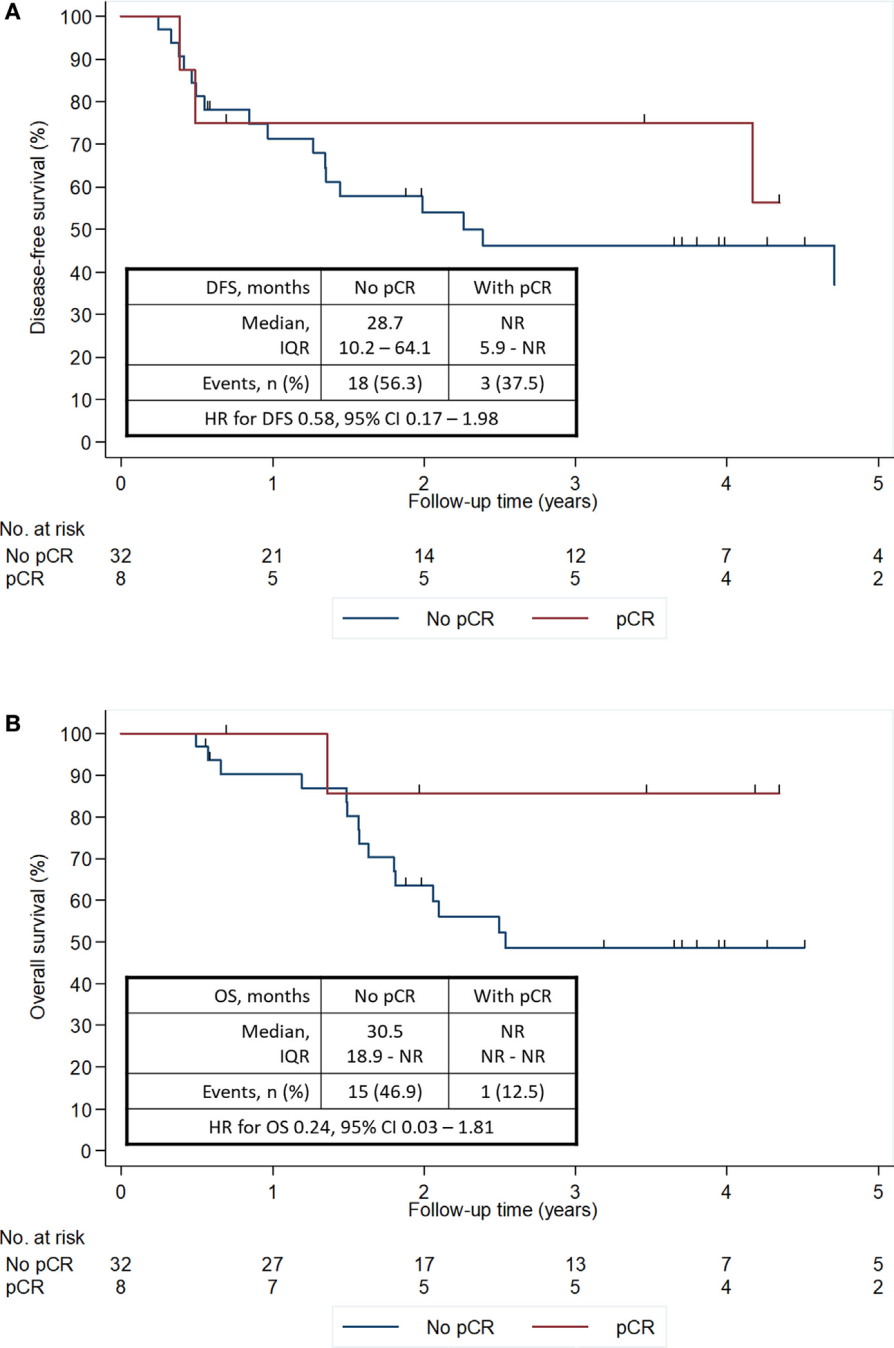
DFS **(A)** and OS **(B)** according to pCR status. CI, confidence interval; DFS, disease-free survival; HR, hazard ratio; IQR, interquartile range; NR, not reached; OS, overall survival; pCR, pathological complete response.

When analyzing survival outcomes according to pCR status, patients reaching a pCR showed a consistent trend for improved outcomes (Figure 3). These results are, however, not significant, with a univariate hazard ratio (HR) of 0.58 (95% CI 0.17–1.98) for DFS and a univariate HR of 0.24 (95% CI 0.03–1.81) for OS. Similarly, when analyzing survival outcomes according to FHBOC, patients with a positive family history showed a trend for improved outcomes. These results are, however, not significant, with a univariate HR of 0.69 (95% CI 0.26–1.81) for DFS and a univariate HR of 0.53 (95% CI 0.17–1.67) for OS.

### Treatment Delivery and Toxicity

Treatment details are summarized in Tables S2 and S3 in Supplementary Material. The majority of patients received six cycles of CAP (*n* = 34, 82.9%). Median relative dose intensity was 95.7% for the overall drug combination. Overall, four patients discontinued treatment due to toxicity, while three patients discontinued treatment due to disease progression (1 extra disease progression was recorded after cycle 6, thus completing all treatments). Median time from last CAP administration to surgery was 6.1 weeks (IQR 5.3–9.4) and the large majority of patients received subsequent adjuvant docetaxel (*n* = 35, 85.4%) and adjuvant radiotherapy (*n* = 37, 90.2%).

Adverse events (AE) are detailed in Tables 4 and 5. The three most common grade 3 or higher AEs were nausea (16.3%), vomiting (14.0%), and neutropenia (9.3%). Febrile neutropenia occurred in three patients (7.0%), of which 1 classified as serious adverse event (SAE). A thromboembolic event (and SAE) was also recorded. AEs of all grades occurring in three or more patients are available in Table S4 in Supplementary Material.

**Table 4:** Adverse events of grade 3 or 4 and serious adverse events (SAEs).

	Grade 3	Grade 4	Total (*n* = 43)	SAEs
Nausea	7	0	7 (16.3)	1
Vomiting	6	0	6 (14.0)	1
Neutrophil count decrease	2	2	4 (9.3)	2
Anemia	4	0	4 (9.3)	1
Fatigue	4	0	4 (9.3)	0
Febrile neutropenia	2	1	3 (7.0)	1
Alopecia	2	0	2 (4.7)	0
Blood glucose increase	2	0	2 (4.7)	0
Diarrhea	2	0	2 (4.7)	0
ALT increase	1	0	1 (2.3)	0
Anorexia	1	0	1 (2.3)	0
Gastrointestinal disorders	0	1	1 (2.3)	1
Hypertension	1	0	1 (2.3)	0
Thromboembolic event	0	1	1 (2.3)	1

**Table 5:** Summary of 10 most common adverse events of any grade.

	AE of any grade, *n* (%)
	Pts receiving CAP (*n* = 43)
Nausea	41 (95.3)
Alopecia	34 (79.1)
Vomiting	34 (79.1)
Fatigue	32 (74.4)
Oral Mucositis	19 (44.2)
Constipation	18 (42.9)
Diarrhea	17 (41.9)
Anorexia	12 (27.9)
Anemia	10 (23.2)
Paronychia	9 (20.9)

## Discussion

In this prospective phase II trial of predominantly stage III patients with TNBC treated with neoadjuvant CAP, 19.5% of patients reached a pCR. After a median follow-up of 4 years, the proportion of patients free of recurrence or death at 3 years was of 51.8%, while the proportion of patients alive at 3 years was of 55.5%. The majority of patients received six cycles of CAP (82.9%) with no new safety signals.

The neoadjuvant treatment of breast cancer has a triple objective: to improve surgical options (ressectability and breast conservation techniques), determine *in vivo* tumor sensitivity to treatment and improve long-term survival outcomes (20). Thus, neoadjuvant chemotherapy is the standard of care for large tumors and pCR an informative biomarker in this setting, given the likelihood to positively impact all the objectives above (7, 21, 22). Despite being less common, pCR is also documented in large tumors and seems as informative, in terms of long-term outcomes, as for smaller tumors (22). In this trial of patients with predominantly stage III TNBC (37/41 patients with stage III tumors), the use of neoadjuvant cisplatin in combination with doxorubicin and cyclophosphamide was associated with a pCR rate of 19.5% (8/41 patients). Despite the large tumor volume at diagnosis, this is a substantial response. Moreover, it is of note that the majority of patients went on to successfully receive surgery with curative intent. These response outcomes are in line with older trials that tested several non-platinum-based regimens in patients with unselected (not exclusively TNBC) and large breast cancers (with 4 cm or more): in this setting, pCR in the breast ranged from 17 to 29% (23–25). Likewise, more recent studies comparing the efficacy of several contemporaneous non-platinum-based regimens, and including tumors as small as 1–2 cm, reported pCR rates ranging from 6 to 26% (26–31). Of note, only in recent times were pCR definitions standardized (22); thus, estimates from previous studies might misrepresent what would be the current pCR rates of such studies.

In TNBC, and more specifically in the basal-like intrinsic subtype, several preclinical studies showed a particular sensitivity to chemotherapy, and especially to DNA-damaging drugs, such as alkylating agents. In this setting, several trials tested the role of neoadjuvant platinum salts in TNBC, obtaining pCR rates ranging from 21 to 61% (8, 13–15, 32, 33). Moreover, recent updates of survival results of GeparSixto trial further showed an 44% improvement in event-free survival (EFS; HR 0.56, 95% CI 0.33–0.96), which translated into an absolute 9.7% improvement in the risk of recurrence at 3 years (76.1 vs. 85.8% for carboplatin-treated patients) (34). A less pronounced, and statistically non-significant, effect of neoadjuvant carboplatin was obtained in the CALGB 40603 trial (EFS HR 0.84, 95% CI 0.58–1.22; 3 years EFS 71 vs. 76% for the carboplatin arm) (35). It is noteworthy that neither GeparSixto nor CALGB 40603 were powered to show a survival difference. In this current trial, after neoadjuvant CAP, the majority of patients received adjuvant docetaxel, and the estimated DFS and OS at 3 years was 51.5 and 55.5%, respectively. These results compare poorly with the trials above; however, they reflect a different patient population, with 32% stage III tumors in the CALGB 40603 trial while 90.2% in the current trial and 59% cN0 in the GeparSixto trial while 22.0% in the current study. Ongoing trials in TNBC, as the NRG-BR003 (NCT02488967) of adjuvant doxorubicin and cyclophosphamide followed by paclitaxel plus or minus carboplatin, and ECOG1131 (NCT02445391) of post-neoadjuvant cisplatin or carboplatin vs. capecitabine in patients with residual disease might further contribute to clarify the role of platinum agents in TNBC.

BRCA status is being studied as a predictive biomarker of response to platinum salts, based on the preclinical evidence that tumors harboring BRCA deficiency are homologous recombination deficient and thus more sensitive to DNA damaging agents, as platinum salts. However, the predictive role of BRCA status is still not clear in this setting. A sub-analysis of GeparSixto showed that response to carboplatin and survival outcomes were independent from germline BRCA (gBRCA) status (34). When comparing pCR rates in patients receiving or not carboplatin, in gBRCA wild-type patients these were of 33.1 vs. 50.8% (OR 2.09, 95% CI 1.24–3.53), respectively, and for gBRCA mutant these were 50.0 vs. 61.5% (OR 1.60, 95% CI 0.52–4.93), respectively. This shows that gBRCA-mutated patients seem to respond more to chemotherapy, but not specifically to addition of carboplatin. Based on these data and the fact that cases of gBRCA mutation tend to cluster in families with known FHBOC, we tested how known FHBOC impacted survival outcomes. Our data show that patients with FHBOC might have an improved recurrence and survival outcomes. A lack of comparator in our trial hinders the possibility to extricate the predictive and/or prognostic information of this particular family history.

The CAP regimen was first developed in ovarian cancer where its safety profile is better established (17–19). In the setting of breast cancer, the concurrent administration of these agents is mostly untested. Such treatment intensification was used as a strategy to maximize response in a group of patients with a large volume of disease. Despite such therapy intensification, no unexpected new safety signals were documented. Nausea (95.3%), vomiting (79.1%), alopecia (79.1%), and fatigue (74.4%) of any grade were the four most common AEs. The tolerability of CAP compares unfavorably with other platinum-based neoadjuvant regimens for breast cancer of less intensified (14) and of more intensified administration of chemotherapy (8). Of note, at the time of the trial design and patient accrual, current drugs for highly emetic regimens, including neurokinin 1 receptor antagonist and olanzapine, were not available. In addition, 3 patients (7.0%) presented febrile neutropenia, of which 1 as a SAE, and paresthesia was present in four patients (9.3%), all grade 1 or 2. The safety profile is comparable to other cisplatin-based neoadjuvant regimens (8, 14). Whether platinum salts should be provided to all, a selected subpopulation, or none of the TNBC; if they should be provided in combination with anthracyclines, taxanes or both; the preferred platinum agent, and the correct dosing/scheduling of these agents are still a matter of active research. These are some of the questions that will continue to shape the coming years of clinical research in this topic.

Furthermore, besides tackling the unmet need of improving outcomes of patients with LABC, a substantial effort should also be allocated to reduce the incidence of such cases. In Brazil, approximately 71% of patients present with stage II/III breast cancer, which contrasts with most westerns countries where between 45 and 55% of patients present with stage II/III tumors (2, 36). Generating the conditions to grant the access to clinical care in settings as Brazil is, therefore, of cornerstone importance.

Despite the interesting findings of this study, it presents several limitations. It is a small, single center trial with no comparison group, which limits the interpretation of the findings. Despite the collection of FHBOC, the specific role of BRCA status and overall homologous recombination deficiency was not addressed in this study. Also, the study was not powered to assess the survival impact of the CAP regimen. Moreover, the 5% pCR rate benchmark used as the null hypothesis for the first stage of the trial, and thus as the rule for early termination, is lower than that usually found in unselected TNBC. However, such cutoff reflects a cohort of predominantly stage III disease, the fact that taxanes were only provided in the adjuvant setting, and previous similar results at the local institution (unpublished data). Finally, as the tumor assessment at diagnosis was performed clinically, the accurate tumor size at diagnosis might have been under or over-estimated.

## Conclusion

Cisplatin in association with doxorubicin and cyclophosphamide was associated with a pCR rate (ypT0ypN0) of 19.5% in a cohort of patients with predominantly stage III tumors. Family history of breast and ovarian cancer did not stratify responders in terms of pCR, but identified patients that might do better in terms of DFS and OS. The tolerability profile of this combination poses clinical challenges to its general use in clinical practice.

## Ethics Statement

This study was carried out in accordance with the recommendations of Comissão Nacional de Ética em Pesquisa with written informed consent from all subjects. All subjects gave written informed consent in accordance with the Declaration of Helsinki. The protocol was approved by the Comissão Nacional de Ética em Pesquisa.

## Author Contributions

AF: study procedures, data analysis, data interpretation, and manuscript writing. OM-F and RS: study procedures, data interpretation, and manuscript writing. JB: study design, study procedures, data interpretation, and manuscript writing.

## Conflict of Interest Statement

The authors declare that the research was conducted in the absence of any commercial or financial relationships that could be construed as a potential conflict of interest.
