# Supplementary material for: Neoadjuvant Treatment of Stage IIB/III Triple Negative Breast Cancer with Cyclophosphamide, Doxorubicin, and Cisplatin (CAP Regimen): A Single Arm, Single Center Phase II Study (GBECAM 2008/02)
Source: Front Oncol. 2018 Jan 24;7:329. doi: 10.3389/fonc.2017.00329 (PMC5787778; doi:10.3389/fonc.2017.00329)
Supplement: Supplementary file 1 [file Data_Sheet_1.docx]

Supplementary table 1 – Final clinical and pathologic tumor size after CAP.

| Final tumor size (mm) on physical examination, n (%)  ≤ 50  >50 - ≤ 70  >70 - ≤ 100  median (IQR)  Missing | 34 (87.2)  3 (7.7)  2 (5.1)  30 (0 – 40)  2 (4.9) |
| --- | --- |
| Final tumor size (mm) on pathology report, n (%)  ≤ 50  >50 - ≤ 70  median (IQR) | 37 (90.2)  4 (9.8)  24 (4 – 38.5) |
| Pathologic T status, n (%)  ypT0  ypT1  ypT2  ypT3  ypT4 | 9 (22.0)  10 (24.4)  16 (39.0)  5 (12.2)  1 (2.4) |
| Pathologic N status, n (%)  ypN0  ypN1  ypN2  ypN3 | 22 (53.7)  4 (9.8)  9 (22.0)  6 (14.5) |
| Pathologic N details  Median (IQR; max) removed nodes  Median (IQR; max) positive nodes | 19 (14 – 24; 34)  0 (0 – 7; 25) |
| DCIS, n (%)  Yes  No | 6 (14.6)  33 (85.4) |

Supplementary table 2 – Cycles performed and dose intensity.

|  | **Patients receiving CAP (n=41)** |
| --- | --- |
| Time from last CAP administration to surgery, weeks  Median  IQR | 6.1  5.3 – 9.4 |
| Adjuvant Docetaxel, n (%) | 35 (85.4) |
| Adjuvant radiotherapy, n (%) | 37 (90.2) |
| Adjuvant radiotherapy dose (Gy)  Median dose  IQR  Missing, n (%) | 50  50 - 50  4 (10.8) |
| Adjuvant radiotherapy fractions, n (%)  25  16  Missing | 30 (93.8)  2 (6.3)  5 (13.5) |

Supplementary table 3 – Other treatment characteristics.

|  | Cyclophosphamide | Doxorubicin | Cisplatin | CAP |
| --- | --- | --- | --- | --- |
| Cycles received, n (%)  6 cycles  5 cycles  4 cycles  3 cycles | 34 (82.9)  4 (9.8)  1 (2.4)  2 (4.9) | 34 (82.9)  4 (9.8)  1 (2.4)  2 (4.9) | 34 (82.9)  4 (9.8)  1 (2.4)  2 (4.9) | 34 (82.9)  4 (9.8)  1 (2.4)  2 (4.9) |
| Dose intensity, mg/m2/3 weeks  Mean  Median  P25-P75  Min  Missing | 466.1  477.8  454.7 – 494.7  312.9  7 (17.1) | 46.7  47.9  45.6 – 49.2  31.2  7 (17.1) | 46.7  47.9  45.6 – 49.2  31.2  7 (17.1) | NA |
| Median relative dose intensity, %  Missing | 95.7  7 (17.1) | 95.7  7 (17.1) | 95.7  7 (17.1) | 95.5  7 (17.1) |

Supplementary table 4 – Adverse events according to CTCAE grade. Only AE with frequency ≥ 3 are shown.

|  | **Grade 1** | **Grade 2** | **Grade 3** | **Grade 4** | **Total**  **(n=43)** |
| --- | --- | --- | --- | --- | --- |
| Alopecia | 7 | 25 | 2 | 0 | 34 (79.1) |
| Anemia | 2 | 4 | 4 | 0 | 10 (23.3) |
| Anorexia | 8 | 3 | 1 | 0 | 12 (27.9) |
| Blood glucose increase | 0 | 2 | 2 | 0 | 4 (9.3) |
| Constipation | 14 | 4 | 0 | 0 | 18 (41.9) |
| Cough | 2 | 1 | 0 | 0 | 4 (9.3) |
| Diarrhea | 12 | 3 | 2 | 0 | 17 (39.5) |
| Dizziness | 4 | 0 | 0 | 0 | 4 (9.3) |
| Dysgeusia | 4 | 2 | 0 | 0 | 6 (14.0) |
| Dysphagia | 4 | 1 | 0 | 0 | 5 (11.6) |
| Esophageal pain | 3 | 0 | 0 | 0 | 3 (7.0) |
| Fatigue | 16 | 13 | 3 | 0 | 32 (74.4) |
| Febrile neutropenia | - | - | 2 | 1 | 3 (7.0) |
| Fever | 5 | 1 | 0 | 0 | 6 (14.0) |
| Headache | 7 | 2 | 0 | 0 | 9 (20.9) |
| Hemoglobin decrease | 2 | 1 | 0 | 0 | 3 (7.0) |
| Insomnia | 5 | 0 | 0 | 0 | 5 (11.6) |
| Myalgia | 4 | 3 | 0 | 0 | 7 (16.3) |
| Nausea | 10 | 24 | 7 | 0 | 41 (95.3) |
| Neutrophil count decrease | 3 | 1 | 2 | 2 | 8 (18.6) |
| Oral mucositis | 10 | 9 | 0 | 0 | 19 (44.2) |
| Pain | 3 | 1 | 0 | 0 | 4 (9.3) |
| Pain in extremity | 2 | 1 | 0 | 0 | 3 (7.0) |
| Paresthesia | 3 | 1 | 0 | 0 | 4 (9.3) |
| Paronychia | 8 | 1 | 0 | 0 | 9 (20.9) |
| Sinusitis | 3 | 0 | 0 | 0 | 3 (7.1) |
| Vaginal inflammation | 2 | 2 | 0 | 0 | 4 (9.3) |
| Vomiting | 11 | 17 | 6 | 0 | 34 (79.1) |
